# Cardiac arrest caused by the application of pituitrin during laparoscopic myomectomy

**DOI:** 10.1186/s12905-023-02255-w

**Published:** 2023-03-18

**Authors:** Jia-Rui Li, Xin Liao, Feng-Qiong Lv, Hui Li

**Affiliations:** 1 Department of Operating Room Nursing, West China, Second University Hospital, Sichuan University/West China School of Nursing, Sichuan University, Chengdu, 610041 Sichuan China; 2 Key Laboratory of Birth Defects and Related Diseases of Women and Children (Sichuan University), Ministry of Education, No. 20 Renmin South Road Section 3, Chengdu, 610041 Sichuan China

**Keywords:** Myomectomy, Pituitrin, Cardiac arrest, Rescue

## Abstract

**Background:**

Pituitrin injection solution is an indispensable hemostatic utilized in clinical practice and is widely used in myomectomy. However, there have been reports of adverse reactions leading to gastrointestinal injury, hyponatremia and hypokalemia, anaphylaxis, cardiac arrest, etc. Thus, the safety of pituitrin should be taken seriously.

**Case presentation:**

In the present study, three cases of cardiac arrest caused by pituitrin injection during laparoscopic myomectomy, who were successfully resuscitated in our hospital, are reported.

**Conclusion:**

The clinical data and surgical procedures in the patient should be analyzed to find the causes of cardiac arrest. Medication and resuscitation should be summarized to ensure the safety of the patient.

## Background

Pituitrin injection solution is a sterile solution extracted from the posterior pituitary gland of animals and contains two active ingredients: oxytocin and vasopressin. These can cause strong contractions of the uterine smooth muscle when injected locally into the uterine body. One study found that the application of pituitrin injection solution into the uterine body during laparoscopic myomectomy was found to promote contractions and reduce hemorrhage [1]. Therefore, pituitrin injection solution is widely used in myomectomy to reduce intraoperative hemorrhage. However, in recent years, severe adverse reactions following the application of pituitrin injection solution have been reported, including gastrointestinal injury, hyponatremia, hypokalemia, osmotic demyelination syndrome, and anaphylaxis. In severe cases, it can lead to changes in vagal and sympathetic tone and cause vagal excitation leading to bradycardia, slowed conduction, sinus bradycardia, cardiac arrest, etc. [2]. Pituitrin injection solution is an indispensable hemostatic in clinical practice, however, problems concerning safety should be considered. The clinical data of three patients who suffered respiratory and cardiac arrest following a pituitrin injection during laparoscopic myomectomy in our hospital are retrospectively analyzed; in addition, the causes of cardiac arrest are analyzed.

## Case presentation

Case No. 1: a female aged 37. Height: 160 cm, bodyweight: 45 kg. The preoperative diagnosis was cervical fibroids. The patient’s general condition was good. The B-ultrasonography revealed that the uterus was morphologically abnormal, approximately 5.9 × 4.3 cm in size. Only a third of the cervix was visible, while the rest was approximately 7.5 × 5.3 cm with hypo-solid echogenicity. The auxiliary examinations were as follows. There was no abnormality in the electrocardiogram (ECG), chest x-ray, or biochemical assays. The vital signs at the entrance of the operation room were as follows: temperature (T): 36.7℃; respirations (R): 20 times/minute; blood pressure (BP): 123/80 mmHg; heart rate (HR): 90 beats/minute. The laparoscopic myomectomy was conducted under general anesthesia at 19:40. The patient was grade I, based on the American Society of Anesthesiologists. The conventional monitoring results were as follows: BP: 128/85 mmHg and HR: 88 beats/minutes. The ECG showed sinus rhythm. At 21:00, the patient was injected with 6 U of pituitrin injection solution (Nanjing Xinbai Pharmaceutical Co., Ltd., 6 U/1 ml), diluted to 6 ml with 0.9% normal saline. One minute later, an ST-segment downward shift was demonstrated in the ECG, followed by an HR of approximately 20 beats/minute. The operation was stopped immediately, and after treatment with intravenous injection of 0.5 mg of atropine and chest compressions, the patient resumed autonomic rhythm at 21:05 with an HR of 115 beats/minute and normal ECG. The surgery was completed at 23:15, and the patient was safely returned to the intensive care unit (ICU) with intensive monitoring. There were no abnormalities in respiratory, circulatory, or cognitive function at the 3-day postoperative follow-up.

Case No. 2: a female aged 32. The preoperative diagnosis was uterine cavity occupancy: residual pregnancy tissue with multiple uterine fibroids. There was no previous medical history. At admission, the patient’s vitals were as follows: T: 36.5℃, P: 76 beats/minute, R: 20 times/minute, BP: 118/80 mmHg, HR: 76 beats/minute. Her height was 158 cm, and her bodyweight was 52 kg. The preoperative examinations were completed, and the results of the B-ultrasonography showed an inhomogeneous and slightly strong echogenicity of 3.0 × 1.8 × 2.6 cm in size, a weak echogenic mass of 4.5 × 3.5 × 4.5 cm in size in the subplasma of the anterior wall, and a weak echogenic mass of 3.6 × 2.9 × 3.4 cm in size and 1.6 cm in diameter in the subplasma of the right wall in the uterine cavity. Sinus rhythm was shown in the ECG with no deviation of the electric axis and no ECG abnormality. A laparoscopic single-site myomectomy was conducted under general anesthesia at 13:00. At 14:13, the patient was intraoperatively injected with 6 U of pituitrin injection solution (Nanjing Xinbai Pharmaceutical Co., Ltd, 6 U/1 ml), diluted to 6 ml with 0.9% of normal saline and injected into the uterine wall. Cardiac arrest occurred in the patient approximately three minutes later. The operation was stopped, and chest compressions were started immediately. Meanwhile, 20 ug of epinephrine and 0.3 mg of atropine were administered intravenously. Sinus rhythm resumed approximately five seconds later with an HR of approximately 45 beats/minute. Then 0.3 mg of atropine was administered intravenously with the continuation of the surgery. The operation ended at 15:25. Vital signs were stable in the patient. There were no abnormalities in respiratory, circulatory, or cognitive function at the 3-day postoperative follow-up.

Case No. 3: a female aged 40 with a preoperative diagnosis of uterine fibroids, scarred uterus, and mild anemia. The past medical history was as follows. Hemorrhoid surgery was conducted more than five years ago, and she had a cesarean section in 2006. Her general condition at admission was as follows: T: 37.2℃, P: 89 beats/minute, R: 20 times/minute, BP: 126/87 mmHg, HR: 89 beats/minute, height: 155 cm, and bodyweight: 62 kg. The preoperative examinations were conducted. The results of the B-ultrasonography showed two to three weakly echogenic masses with a size of 4.6 × 3.0 × 4.1 cm and clear borders between the muscular walls and subplasma. The ECG results showed sinus rhythm, no deviation of the electric axis, and no ECG abnormality. A laparoscopic myomectomy was conducted under general anesthesia at 11:00. At 11:38, the patient was injected with 6 U of pituitrin injection solution intraoperatively (Nanjing Xinbai Pharmaceutical Co., Ltd, 6 U/1 ml), diluted to 6 ml with 0.9% of normal saline. The pituitrin was injected into the uterine wall and avoid undesirable systemic effects of inadvertent intravascular injection. Approximately two minutes later, the ECG waveform disappeared in the monitor. The surgical operation was immediately stopped with a pause of CO_2_ infusion and a partial expulsion of CO_2_ to reduce the abdominal pressure. Chest compressions were conducted, and 40 ug of epinephrine and 0.5 mg of atropine were administered intravenously. The heartbeat resumed after five seconds, and sinus rhythm was restored after 30 s with a BP of 113/74 mmHg. The operation was completed at 13:20, and ICU monitoring continued for 24 h after extubation until the vital signs were stable. The patient was transferred to the general ward with clear consciousness. There were no abnormalities in respiratory, circulatory, or cognitive function at the 3-day postoperative follow-up.

## Discussion and conclusion

The changes in BP, HR, and T should be closely observed intraoperatively. Because pituitrin can affect HR, BP, and body temperature [3] in the case of pituitrin injection, the dynamic changes in ECG and BP in the patient should be closely observed to discover the changes in the condition and deal with the problems in time to provide a safety guarantee for the operation. Pituitrin can cause vasoconstriction, resulting in increased peripheral resistance and higher BP. This can lead to the excitation of pressure receptors, increased cardiac vagal efferent impulses, and decreased cardiac sympathetic efferent impulses and reduced cardiac output. These can result in slowed HR or even cardiac arrest. The authors in the present study suggested that the anesthesiologist should be reminded before injecting pituitrin to deepen the anesthesia appropriately. This might control the patient’s BP and inhibit the excitation of the pressure receptors, thus preventing or reducing the occurrence of adverse reactions in patients after the application of pituitrin. Meanwhile, for patients undergoing myomectomy, care for the perioperative temperature should be conducted: room temperature set at 24 °C–26℃, the application of disposable heating blankets, and warmed flushing solution.

Intraoperative dosing and injection of pituitrin should be improved. Clinically, the local injection of pituitrin is sufficient to enhance uterine contractions and reduce hemorrhage, but different doses of pituitrin may have different effects on the circulatory system [4] . Inconsistent with typically 20 units of vasopressin are diluted in 100 mL saline, pituitrin was administered as a single injection of 6 U (dissolved in 5 ml of normal saline) in the present study. Therefore, the high dose of vasopressin solution may be one reason for the cardiovascular complications. Previous study suggested that fractionated, small doses of pituitrin might effectively reduce intraoperative hemorrhage and stabilize the intraoperative BP in patients undergoing laparoscopic uterine fibroid debridement [5] . Therefore, pituitrin should be injected intraoperatively with a small amount in each of the two fractionated doses.

The puncture needle for intraoperative administration of pituitrin should be improved. Most of the pituitrin puncture needles adopted in our hospital’s department were laparoscopic puncture needles, which were too long and had too thick a diameter. Thus, it was not easy to observe the blood return when puncturing the vessels, and the dose might not be accurate enough and affect the efficacy of the drug. A lumbar puncture needle or a common syringe (5–10 ml in size) could be used to puncture at 2–3 cm above the median mons pubis and reduce the pneumoperitoneal pressure before puncture to facilitate puncture and observation of blood return.

Adequate emergency drugs and apparatus should be prepared before surgery. The commonly used cardiovascular emergency drugs such as epinephrine and atropine should be routinely prepared before surgery and diluted in proportion in advance so that these drugs can quickly be administered intravenously in the event of an accident. Apparatus such as defibrillators should be checked in advance for performance and be in a standby condition. For the three cases in the present study, the resuscitation progressed well and successfully due to the well-prepared drugs and apparatus.

In conclusion, administering pituitrin in patients undergoing myomectomy might be a double-edged sword, possibly leading to decreased HR or even cardiac arrest in severe cases. These conditions might progress rapidly, making them critical. During the operation, close attention should be paid to the vital signs to identify changes in BP, HR, and the waveforms in the ECG.

## Data Availability

The datasets used and/or analysed during the current study available from the corresponding author on reasonable request.

## Abbreviations

ECG: Electrocardiogram
T: Temperature
R: Respirations
BP: Blood pressure
HR: Heart rate
ICU: Intensive care unit
